# Genomic investigation of the coronavirus disease-2019 outbreak in the Republic of Korea

**DOI:** 10.1038/s41598-021-85623-6

**Published:** 2021-03-16

**Authors:** Jeong-Min Kim, Sung Yong Park, Daesang Lee, Jun-Sub Kim, Youngjoon Park, Jin Gwack, Mi Young Kim, Dong Hyun Song, Seong Tae Jeong, Yoon-Seok Chung, Cheon Kwon Yoo, Ha Youn Lee, Myung-Guk Han

**Affiliations:** 1 Bureau of Infectious Disease Diagnosis Control, Korea Disease Control and Prevention Agency, 187 Osongsaengmyeong2-ro, Osong-eup, Heungdeok-gu, Cheongju-si, 28159 Republic of Korea; 2 Department of Molecular Microbiology and Immunology, Keck School of Medicine, University of Southern California, Los Angeles, CA 90089-9605 USA; 3 The 4Th R&D Institute, Agency for Defense Development, Yuseong, Daejeon, 34186 Republic of Korea; 4 Director for Epidemiological Investigation Analysis, Korea Disease Control and Prevention Agency, 187 Osongsaengmyeong2-ro, Osong-eup, Heungdeok-gu, Cheongju-si, 28159 Republic of Korea; 5 Division of Emerging Infectious Disease Response, Bureau of Infectious Disease Emergency Preparedness and Response, Korea Disease Control and Prevention Agency, 187 Osongsaengmyeong2-ro, Osong-eup, Heungdeok-gu, Cheongju-si, 28159 Republic of Korea; 6 Division of Infectious Disease Response, Capital Regional Center for Disease Control and Prevention, Korea Disease Control and Prevention Agency, 187 Osongsaengmyeong2-ro, Osong-eup, Heungdeok-gu, Cheongju-si, 28159 Republic of Korea

**Keywords:** Genetics, Molecular biology, Diseases

## Abstract

The South Korean government effectively contained the coronavirus disease-2019 (COVID-19) outbreak primarily associated with a religious group. We conducted SARS-CoV-2 whole genome sequencing of 66 cases to investigate connections among the initial South Korean cases and the religious group outbreak. We assessed the accuracy of genomic investigation by comparing the whole genome sequences with comprehensive contact tracing records. Five transmission clusters were estimated among the 15 initial cases. The six close-contact cases and two potential exposure pairs identified by contact tracing showed two or fewer nucleotide base differences. Additionally, we identified two transmission clusters that were phylogenetically distinct from the initial clusters, sharing common G11083T, G26144T, and C14805T markers. The strain closest to the two additional clusters was identified from a pair of identical sequences isolated from individuals who traveled from Wuhan to Italy. Our findings provide insights into the origins of community spread of COVID-19.

## Introduction

**Figure 1:**
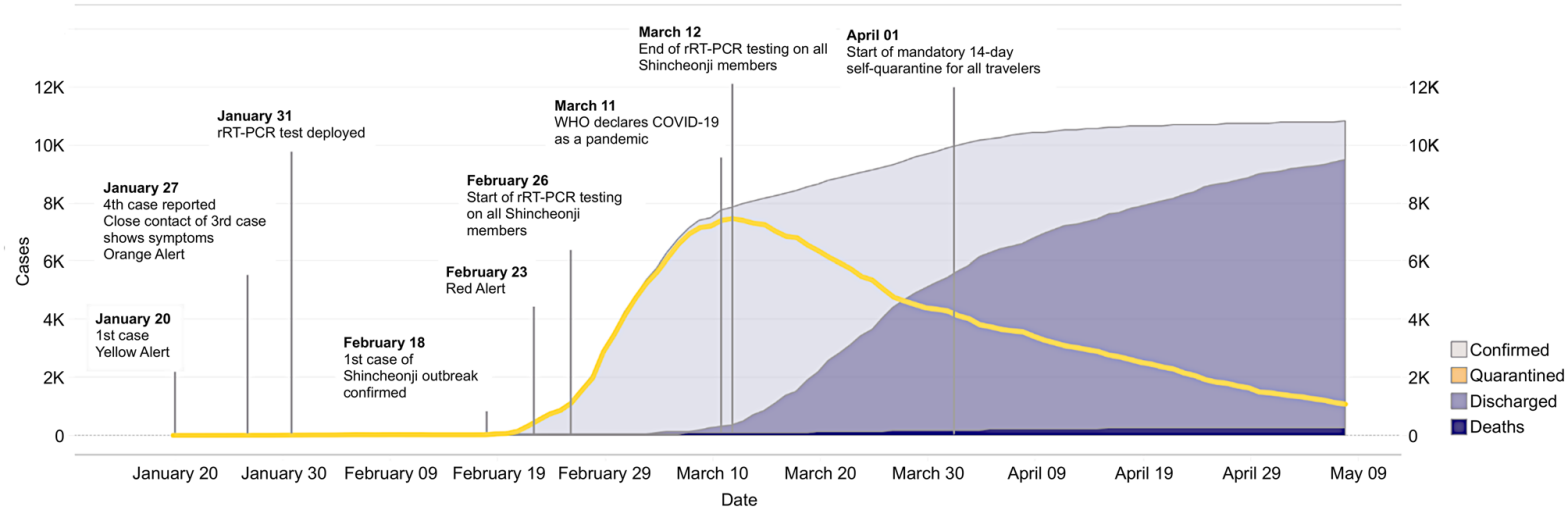
South Korea’s COVID-19 outbreak and intervention timeline.

After identifying a massive outbreak of coronavirus disease-2019 (COVID-19) primarily within a religious group on February 18, 2020, the South Korean government immediately began contact tracing and mandated self-isolation for all members of the church (~ 240,000 individuals). As a result, the government was able to halt the spread of the virus at approximately 10,000 cases for more than 1 month (Fig. 1). Notably, the government of South Korea did not issue a country-wide stay at home order and only concentrated on individuals specifically identified through contact tracing.

In retrospect, the prompt responses of the South Korean government were effective in containing the COVID-19 spread. However, this method required extensive in-person questionnaires that not only were susceptible to human error but were also often met with resistance owing to privacy concerns^1^. Herein, we propose performing genomic investigation as a supplementary tool to epidemiological contact tracing, which could help determine the current and future spread of the disease. With genomic investigation, we could detect the source of infection directly from the patient’s viral genotype and reduce uncertainties in transmission route identification, thereby guiding more effective intervention implementations^2–5^. In this study, we evaluated the accuracy of genomic investigation by directly comparing South Korea’s comprehensive contact tracing data with the genomic associations among severe acute respiratory syndrome coronavirus 2 (SARS-CoV-2) whole genome sequences.

## Results

### South Korea COVID-19 outbreak and response

When the Wuhan government first reported 27 cases of pneumonia on December 31, 2019, the South Korean government immediately issued the lowest threat alert (blue) and started to screen air travelers from Wuhan using infrared thermometers and a pan-corona virus PCR kit. Upon discovery of the first confirmed case on January 20, the government promptly raised the threat level to yellow and subsequently to orange on January 27 following the identification of a fourth case (Fig. 1). By February 17, 30 cases were identified, and 10 had already recovered. Additionally, 11 cases were confirmed to be caused by close contact, and 19 were confirmed to be imported.

The first instance of a massive outbreak in the church was identified on February 18, 2020. This case was a member of the church without any international travel records. Ten close contacts of this individual, mainly including church members, were confirmed on the following day, and positive cases from the church subsequently increased rapidly (Fig. 1). On February 20, another outbreak was identified at a hospital complex, and subsequently, the first COVID-19-related death was reported. By February 22, the total number of cases rose to 346, among which 169 (48.8%) and 108 (31.2%) cases were related to the church outbreak and the regional hospital outbreak, respectively.

The government started screening all church members in Daegu and all individuals at the hospital complex. When the cases rose to 600 on February 23, the government raised the alert level to red, officially declaring a national emergency (Fig. 1). On February 25, the Korean Government started massive contact tracing and real-time RT-PCR testing on the church members in South Korea, completing around 240,000 tests in just 16 days by March 12 (Fig. 1). The resulting exponential increase in cases, however, led to a shortage of negative pressure isolation rooms and overall burden on the South Korean healthcare system. Following this peak, there were several minor outbreaks related to the known cases, but all were successfully contained owing to rapid screening efforts and preemptive response measures for vulnerable groups. Additionally, instances continued to be imported, particularly from Europe and North America, and travel-related cases became greater than 10% of the total daily confirmed cases on March 31. In response, the government enforced a mandatory 14-day self-quarantine for all travelers on April 1 (Fig. 1). On May 8, one local case was identified, whereas 11 were deemed imported.

### Demographics of patients with COVID-19

**Figure 2:**
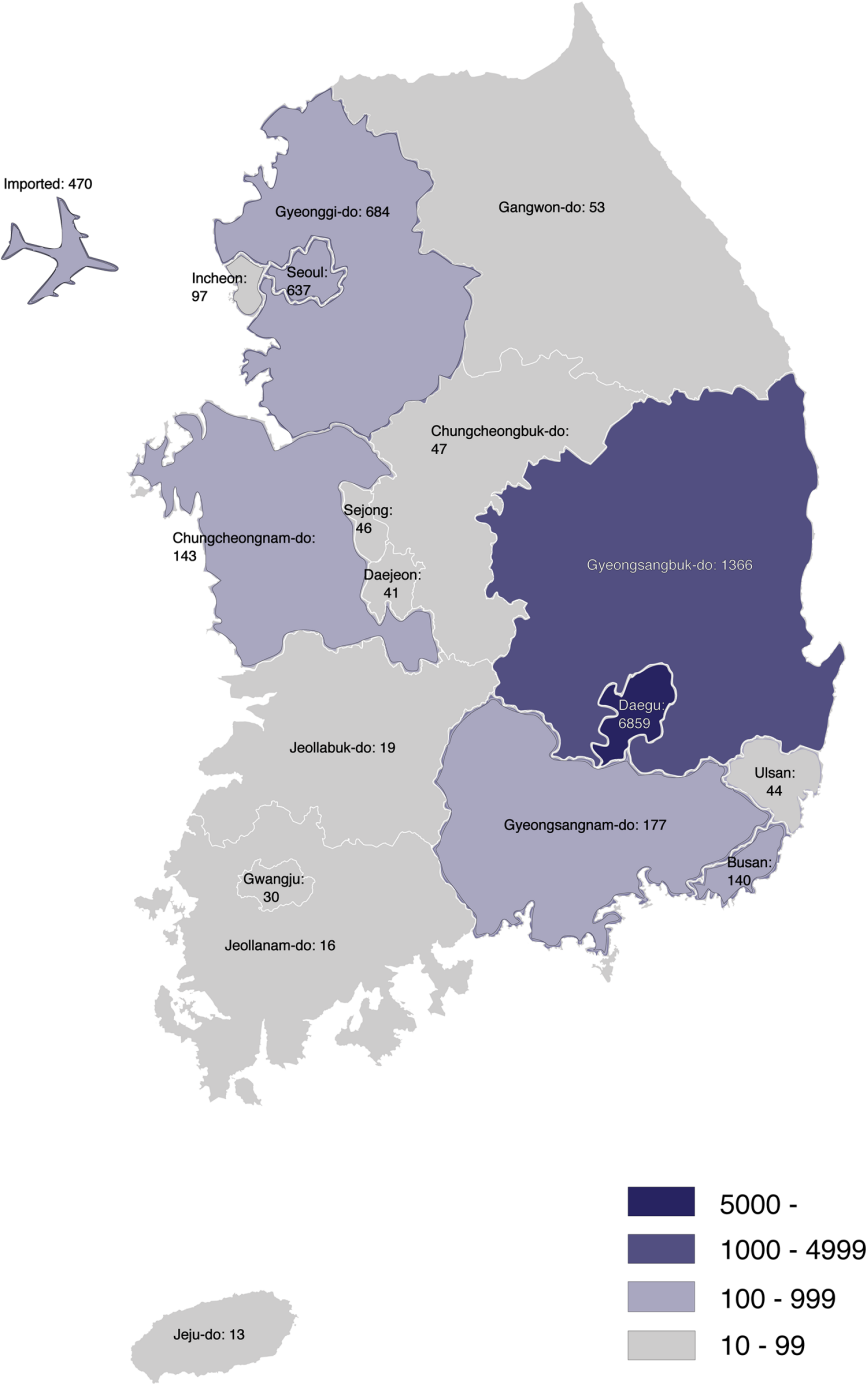
Regional distribution of COVID-19 cases by May 8, 2020 in South Korea. The majority of cases (6859 of 10,822 cases) occurred in Daegu (dark blue), and the total number of imported cases (marked by the airplane) was 470.

As of May 8, approximately 60% of total confirmed cases were female; however, the mortality rate was higher in males than in females (3.0% versus 1.9%, respectively; Table 1). Mortality rates also increase with age (0% and 1% for individuals below 30 years old and below 60 years old, respectively; 25% for individuals 80 years old and older). Additionally, 80.5% of total cases were part of local cluster outbreaks, and 10.8% were imported. Notably, 5212 confirmed cases related to the church outbreak were localized around Daegu (dark blue in Fig. 2), accounting for 48.1% of total confirmed cases in South Korea.

**Table 1:** Demographics and mortality rate of 10,840 confirmed cases of COVID-19 in South Korea by May 8, 2020.

	Confirmed cases	Deaths	Mortality rate (%)
Total	10,840	256	2.4
**Sex**			
Male	4406 (40.7%)	133 (52.0%)	3.0
Female	6434 (59.4%)	123 (48.1%)	1.9
**Age (years)**			
80 and above	488 (4.5%)	122 (47.7%)	25.0
70–79	710 (6.6%)	77 (30.1%)	10.9
60–69	1355 (12.5%)	37 (14.5%)	2.7
50–59	1958 (18.1%)	15 (5.8%)	0.77
40–49	1438 (13.3%)	3 (1.2%)	0.21
30–39	1177 (10.9%)	2 (0.8%)	0.17
20–29	2979 (27.5%)	0	0
10–19	594 (5.5%)	0	0
0–9	141 (1.3%)	0	0

### Genomic investigation cohort

We performed SARS-CoV-2 whole genome sequencing on 66 cases to investigate associations between the church outbreak and the initial cases within South Korea (Table 2). Short-read high-throughput sequencing was performed for whole genome coverage. The total number of filtered reads, number of reads mapped to the reference genome (Wuhan-Hu-1, NCBI Reference Sequence: NC_045512, GenBank accession number: MN908947.3), base coverage, and average sequencing depth are presented in Supplementary Table S1. As detailed in Table 2, we obtained viral sequences from (i) 15 initial cases (out of 30) prior to the church outbreak, (ii) 37 cases (out of 311) within the first 5 days of the church outbreak, and (iii) 14 other cases presumed to be related to the church outbreak by contact tracing records.

**Table 2:** Specimen collection date, cluster assignment and mutations from Wuhan-Hu-1 of COVID-19 cases in South Korea whose SARS-CoV-2 whole-genome sequences were reported in this study.

Case ID	Specimen collection date	Cluster assignment	Mutations from Wuhan-Hu-1
5174*	19-Jan-2020	C1	G26144T(ORF3a-G251V)
7156*	30-Jan-2020	C1	None
1534^§^	1-Feb-2020	C1	None
7834	5-Feb-2020	C1	None
3542*	25-Jan-2020	C2	T4402C, G5062T(NSP3-L781F), C8782T, T28144C (ORF8-L84S)
5431*	30-Jan-2020	C2	T4402C, G5062T(NSP3-L781F), C8782T, T28144C(ORF8-L84S)
9273*	30-Jan-2020	C2	C1779T (NSP2-S325F), T4402C, G5062T(NSP3-L781F), C8782T, C15017T(NSP12-A526V), T28144C(ORF8-L84S)
4536*	2-Feb-2020	C2	C4276T, T4402C, G5062T(NSP3-L781F), C8782T, T28144C(ORF8-L84S)
2314^§^	4-Feb-2020	C2	T4402C, G5062T(NSP3-L781F), C8782T, C11572, T28144C(ORF8-L84S)
4023^§^	5-Feb-2020	C2	T4402C, G5062T(NSP3-L781F), C8782T, C17474T(NSP13-T413I), T28144C(ORF8-L84S)
6303^§^	18-Feb-2020	C2	T4402C, G5062T(NSP3-L781F), C8782T, T28144C(ORF8-L84S)
6712	21-Feb-2020	C2	T4402C, G5062T(NSP3-L781F), C8782T, C21846T(S-T95I), T22117G(S-N185K), T28144C(ORF8-L84S)
3401	6-Feb-2020	C3	T3086C(NSP3-F123L), C6255T(NSP3-A1179V), C8782T, G11083T(NSP6-L37F), G17122A(NSP13-A296T), T28144C(ORF8-L84)
7134^§^	30-Jan-2020	C4	A1740C(NSP2-K312T), T8767C, C8782T, C17104T(NSP13-H290Y), G26167T(ORF3a-V259L), T28144C(ORF8-L84S)
8423*	30-Jan-2020	C4	G7675T, T8767C, C8782T, C17104T(NSP13-H290Y), C18167T(NSP14-P43L), G26167T(ORF3a-V259L), T28144C(ORF8-L84S)
7098^§^	8-Feb-2020	C5	C1101T(NSP2-S99F), G3119A(NSP3-E134K), C8782T, C24034T, T26729C, G28077C(ORF8-V62L), T28144C(ORF8-L84S)
8123	9-Feb-2020	C5	C1101T(NSP2-S99F), G3119A(NSP3-E134K), C8782T, C24034T T26729C, C26873T, G28077C(ORF8-V62L), T28144C(ORF8-L84S)
9234	20-Feb-2020	C5	C1101T(NSP2-S99F), G3119A(NSP3-E134K), C8782T, C24034T T26729C, G28077C(ORF8-V62L), T28144C(ORF8-L84S)
2310	17-Feb-2020	C6	G5572T(NSP3-M951I), G11083T(NSP6-L37F), C14805T, G26144T(ORF3a-G251V)
7450	18-Feb-2020	C6	A2406G(NSP2-K534R), G5572T(NSP3-M951I), G11083T(NSP6-L37F), C14805T, G26144T(ORF3a-G251V)
2383	18-Feb-2020	C6	G5572T(NSP3-M951I), G11083T(NSP6-L37F), C14805T, T15399C, G26144T(ORF3a-G251V)
8923	18-Feb-2020	C6	G5572T(NSP3-M951I), G11083T(NSP6-L37F), C14805T, G26144T(ORF3a-G251V)
2305	18-Feb-2020	C6	G5572T(NSP3-M951I), G11083T(NSP6-L37F), C14805T, G26144T(ORF3a-G251V)
3424	18-Feb-2020	C6	G5572T(NSP3-M951I), G11083T(NSP6-L37F), C14805T, G26144T(ORF3a-G251V)
2069	18-Feb-2020	C6	C3411T(NSP3-A231V), G5572T(NSP3-M951I), G11083T(NSP6-L37F), C13551T, C14805T, G26144T(ORF3a-G251V)
1126	19-Feb-2020	C6	T3142C, G5572T(NSP3-M951I), G11083T(NSP6-L37F), C14805T, G26144T(ORF3a-G251V)
7092	19-Feb-2020	C6	T921A(NSP2-L39Q), G5572T(NSP3-M951I), G11083T(NSP6-L37F), C14805T, C15216T, G26144T(ORF3a-G251V)
4528	19-Feb-2020	C6	T693C(NSP1-F143S), G5572T(NSP3-M951I), G11083T(NSP6-L37F), C14805T, G26144T(ORF3a-G251V)
9340	19-Feb-2020	C6	G5572T(NSP3-M951I), G11083T(NSP6-L37F), C14805T, G26144T(ORF3a-G251V)
3495	19-Feb-2020	C6	G5572T(NSP3-M951I), G11083T(NSP6-L37F), C14805T, G26144T(ORF3a-G251V)
5253	20-Feb-2020	C6	G5572T(NSP3-M951I), G11083T(NSP6-L37F), C14805T, G26144T(ORF3a-G251V)
1209	19-Feb-2020	C6	G5572T(NSP3-M951I), G11083T(NSP6-L37F), C14805T, G26144T(ORF3a-G251V)
9061	19-Feb-2020	C6	G5572T(NSP3-M951I), G11083T(NSP6-L37F), C14805T, C21575T(S-L5F), G26144T(ORF3a-G251V)
3470	19-Feb-2020	C6	G5572T(NSP3-M951I), G11083T(NSP6-L37F), C14805T, G26144T(ORF3a-G251V)
4587	19-Feb-2020	C6	G5572T(NSP3-M951I), C11074T, G11083T(NSP6-L37F), C12049T, C14805T, A17457R, T25219C, G26144T(ORF3a-G251V)
1284	19-Feb-2020	C6	G5572T(NSP3-M951I), G11083T(NSP6-L37F), C14805T, G26144T(ORF3a-G251V)
9023	19-Feb-2020	C6	G5572T(NSP3-M951I), G11083T(NSP6-L37F), C14805T, G21778A, T22928C(S-F456L), C23917T, G26144T(ORF3a-G251V)
2301	19-Feb-2020	C6	T3142C, G5572T(NSP3-M951I), G11083T(NSP6-L37F), C14805T, G26144T(ORF3a-G251V)
4327	19-Feb-2020	C6	G5572T(NSP3-M951I), C11074T, G11083T(NSP6-L37F), C12049T, C14805T, G26144T(ORF3a-G251V)
5028	21-Feb-2020	C6	G5572T(NSP3-M951I), G11083T(NSP6-L37F), C14805T, T15570N C16575T, A18095G(NSP14-H19R), G20974T(NSP16-D106Y), T23395A, G26144T(ORF3a-G251V)
7821	20-Feb-2020	C6	G5572T(NSP3-M951I), G11083T(NSP6-L37F), C14805T, G22955T(S-E465stop), G26144T(ORF3a-G251V)
3460	20-Feb-2020	C6	G5572T(NSP3-M951I), A9889G, G11083T(NSP6-L37F), C14805T, G26144T(ORF3a-G251V)
6314	20-Feb-2020	C6	T921A(NSP2-L39Q), G5572T(NSP3-M951I), G11083T(NSP6-L37F), C14805T, G26144T(ORF3a-G251V)
3407	21-Feb-2020	C6	G5572T(NSP3-M951I), C6327T(NSP3-T1203I), G11083T(NSP6-L37F), C14805T G26144T(ORF3a-G251V)
1276	21-Feb-2020	C6	G5572T(NSP3-M951I), G11083T(NSP6-L37F), C14805T, G26144T(ORF3a-G251V)
2697	21-Feb-2020	C6	G5572T(NSP3-M951I), G11083T(NSP6-L37F), C14805T, G26144T(ORF3a-G251V)
3058	21-Feb-2020	C6	G5572T(NSP3-M951I), G11083T(NSP6-L37F), C14805T, G26144T(ORF3a-G251V)
2840	23-Feb-2020	C6	G5572T(NSP3-M951I), C6267T(NSP3-A1183V), G11083T(NSP6-L37F), C14805T, G26144T(ORF3a-G251V)
3486	21-Feb-2020	C6	G5572T(NSP3-M951I), G11083T(NSP6-L37F), C14805T, C19983T, G26144T(ORF3a-G251V)
2397	22-Feb-2020	C6	G5572T(NSP3-M951I), G11083T(NSP6-L37F), C14805T, G26144T(ORF3a-G251V)
1037	20-Feb-2020	C6	G5572T(NSP3-M951I), G11083T(NSP6-L37F), C14805T, C19983T, G26144T(ORF3a-G251V)
3479	23-Feb-2020	C6	G5572T(NSP3-M951I), A9889G, G11083T(NSP6-L37F), C14805T, G26144T(ORF3a-G251V)
2058	22-Feb-2020	C6	G5572T(NSP3-M951I), G11083T(NSP6-L37F), T12520C, C14805T G26144T(ORF3a-G251V)
5679	24-Feb-2020	C6	G5572T(NSP3-M951I), A9889G, G11083T(NSP6-L37F), C14805T, C16961T(NSP13-P242L), G26144T(ORF3a-G251V)
7239	26-Feb-2020	C6	G5572T(NSP3-M951I), G11083T(NSP6-L37F), C14805T, G26144T(ORF3a-G251V), C28311T(N-P13L)
2205	20-Feb-2020	C7	C1342A(NSP2-Y179stop), G11083T(NSP6-L37F), C14805T, G26144T(ORF3a-G251V), C26681T
1297	20-Feb-2020	C7	G11083T(NSP6-L37F), C14805T, G26144T(ORF3a-G251V), C26681T
2307	20-Feb-2020	C7	G11083T(NSP6-L37F), C14805T, G26144T(ORF3a-G251V), C26681T
2395	20-Feb-2020	C7	G11083T(NSP6-L37F), C14805T, A23512G, C25416T, G26144T(ORF3a-G251V), C26681T
2352	20-Feb-2020	C7	G11083T(NSP6-L37F), C14805T, G26144T(ORF3a-G251V), C26681T
2386	20-Feb-2020	C7	G11083T(NSP6-L37F), C14805T, T21769C, G26144T(ORF3a-G251V), C26681T
1034	20-Feb-2020	C7	G11083T(NSP6-L37F), C14805T, G26144T(ORF3a-G251V), C26681T, G27556T(ORF7a-A55S)
2356	20-Feb-2020	C7	G11083T(NSP6-L37F), C14805T, G26144T(ORF3a-G251V), C26681T
3980	20-Feb-2020	C7	G11083T(NSP6-L37F), C14805T, G26144T(ORF3a-G251V), C26681T
2389	20-Feb-2020	C7	G11083T(NSP6-L37F), C14805T, G26144T(ORF3a-G251V), C26681T
7823	21-Feb-2020	C7	C11074T, G11083T(NSP6-L37F), C14805T, G26144T(ORF3a-G251V), C26681T

Sequences from unmarked cases were obtained directly from patient NP/OP specimens.

*NA* not available.

*Sequence obtained via the viral cell culture method.

^§^Sequence obtained from both patient NP/OP specimens and the viral cell culture method.

### Initial cases

**Figure 3:**
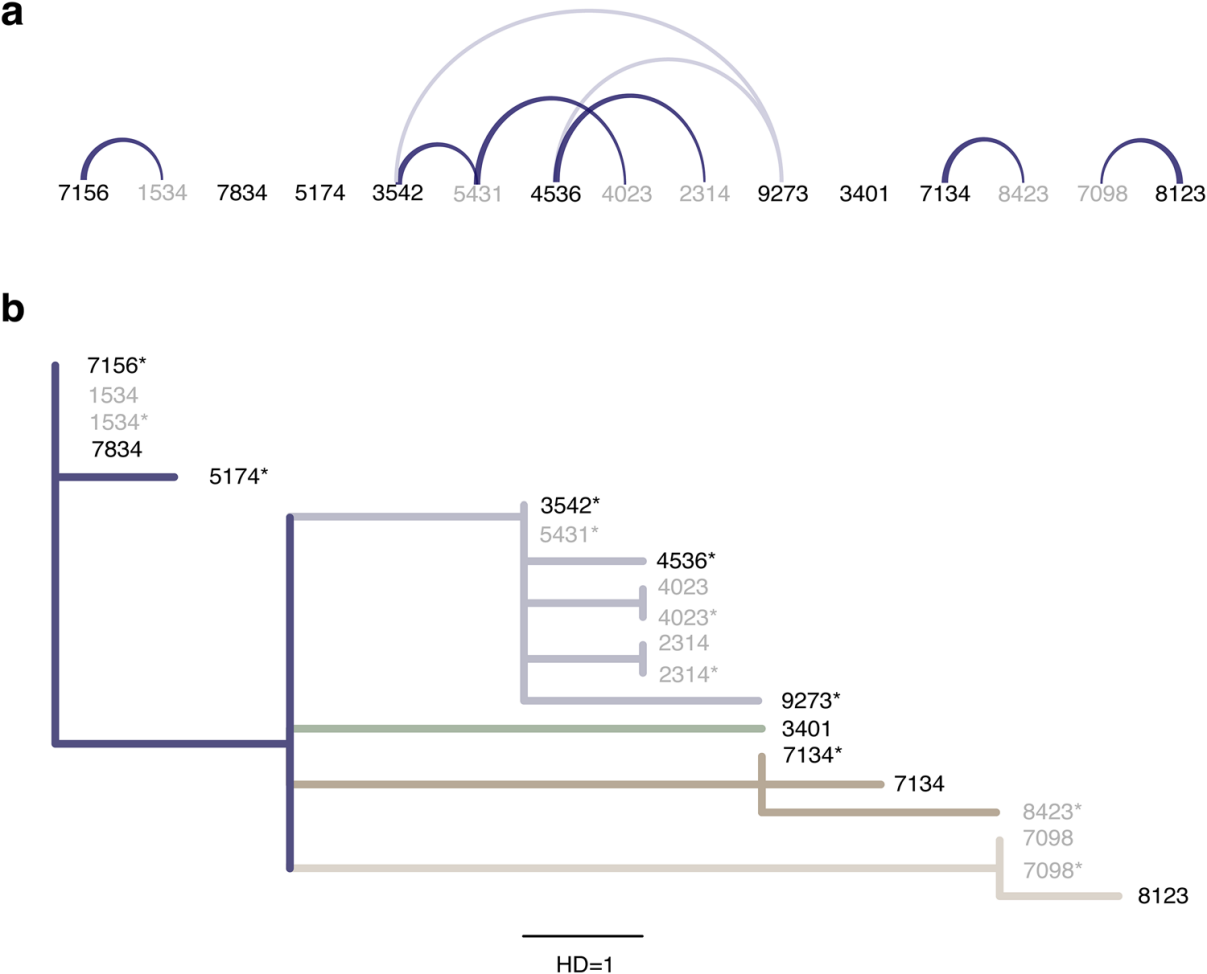
Contact tracing record arc diagram (**a**) and maximum likelihood tree (**b**) of initial 15 cases prior to a church outbreak in South Korea. (**a**) Confirmed close contacts via epidemiological tracing are linked with a solid curve, with the transmission directionality (from case 8123 to case 7098, for example) indicated by width (wide: source; narrow: recipient). Potentially exposed pairs of individuals (within the same relative area) are marked with faded curves (for example, between Case 4536 and Case 9273). Imported (local) cases are presented by solid (faded) numbers. (**b**) Five transmission clusters inferred via maximum likelihood tree analysis on the SARS-CoV-2 whole genome sequences from the 15 cases: from top to bottom, cluster 1 (blue), cluster 2 (light blue), cluster 3 (green), cluster 4 (brown), cluster 5 (light brown). A star denotes sequences obtained by virus inoculation and cell culture. The length of the black bar indicates a Hamming distance (HD) of 1 or a one nucleotide base difference. The branch support values of the tree using the SH-like likelihood ratio test were estimated as around 1.

Our genomic investigation estimated five transmission clusters among the 15 cases prior to the church outbreak (Fig. 3). The first cluster (C1) contained four cases, i.e., cases 5174, 7156, 1534, and 7834, where all but case 5174 showed sequences identical to that of one of the earliest cases (Wuhan-Hu-1 strain: NCBI Reference Sequence: NC_045512, GenBank accession number: MN908947.3), and case 5174 harbored one nonsynonymous mutation (G26144T)^6^. This aligned with the reported contact tracing results (Fig. 3a), as follows: (i) case 7156 had interacted with a Wuhan resident and was a family member of case 1534; (ii) case 7834 had met a confirmed case from Wuhan, and (iii) case 5174 was from Wuhan. Taken together, all four individuals in C1 were either closely linked to Wuhan or had close contact of Wuhan cases, revealing a strong correlation between our genomic investigation and the epidemiological tracing.

The second cluster (C2) consisted of cases 9273, 3542, 5431, 4536, 2314, a and 4023 (Fig. 3b). Case 9273 had been working in Wuhan when case 3542 had visited, and the sequence of the virus from case 9273 showed two mutations compared with case 3542 and case 5431, but shared two common mutations from the C1 founder sequence. Case 3542 had met case 5431, and the genomic sequences of the virus for these cases were identical. Case 4023 was a close-contact of Case 5431, with the sequences showing a difference of only one mutation. Case 4536 had worked in Wuhan prior to diagnosis in South Korea and interacted with case 2314; the genomic sequence of the virus from case 4536 showed one mutation compared with that of case 3542, whereas cases 4536 and 2314 had two nucleotide substitutions. Therefore, all confirmed close-contact cases had either identical sequences or sequence pairs with one nucleotide difference, whereas potential exposure pairs showed only two mutations.

The three other sequence clusters were also consistent with contact tracing data (Fig. 3). The third cluster (C3) has only one member, Case 3401, a Wuhan resident who did not have close contact with any of the other cases in South Korea. The fourth (C4) and fifth (C5) clusters each consisted of two members, who shared six and eight common mutation markers from the first cluster, respectively. Thus, our maximum likelihood tree constructed from mutation signatures was consistent with epidemiological contact tracing records, demonstrating the effectiveness of genomic sequencing in discerning close-contact transmission clusters.

### Church outbreak analysis

**Figure 4:**
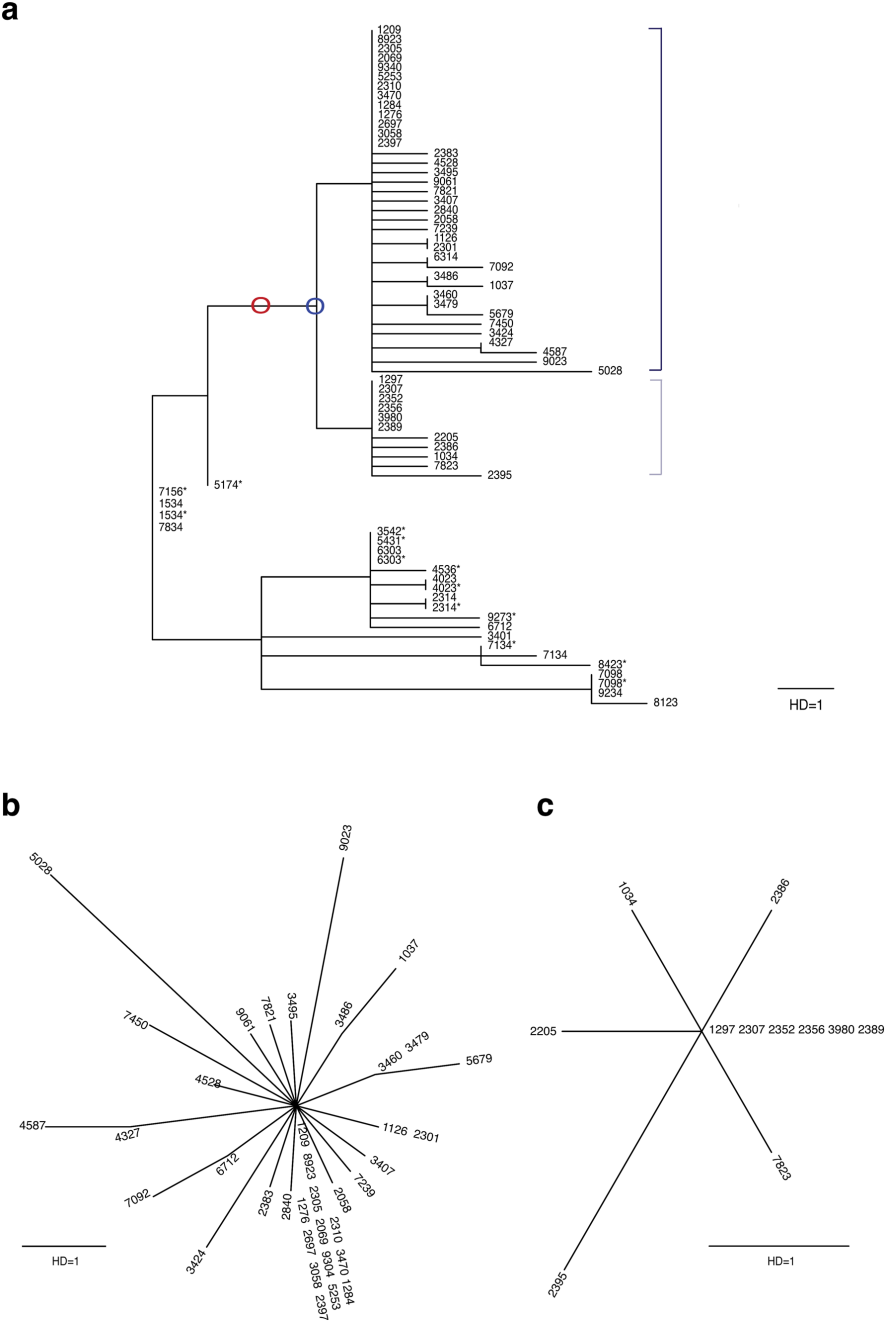
Maximum likelihood tree of all 66 cases. (**a**) Maximum likelihood tree of all 66 South Korean cases that were sequenced. The church cluster (cluster 6) is marked by blue brackets, and the hospital cluster (cluster 7) is marked by light blue. The branch support values of the tree using the SH-like likelihood ratio test were estimated as around 1. The most recent common ancestor sequence of clusters 6 and 7 is presented by blue circle and the strain that shared two common mutation markers with this ancestor is presented by red circle. (**b**) Star-like phylogeny of the 37 church cluster cases (cluster 6, dark blue in (**a**)) (**c**) Star-like phylogeny of the 11 hospital cluster cases (cluster 7, light blue in (**a**)).

Figure 4 shows the maximum likelihood tree of 51 cases after the onset of the church outbreak alongside the 15 initial cases that were sequenced. Most cases after the onset of the outbreak were phylogenetically distinct from the prior cases and formed two closely related, yet independent clusters: C6 (Fig. 4a, dark blue brackets), which mainly consisted of church members in Daegu; and C7 (Fig. 4a, light blue brackets), which consisted of all and only cases from the hospital.

Additionally, both clusters displayed star-like phylogeny (Fig. 4b,c), a signature of random neutral evolution^7^. Both clusters had multiple identical sequences at the tree center (13 out of 37 for cluster 6 and six out of 11 for cluster 7), an evolutionary pattern frequently observed in acute-stage human immunodeficiency virus sequences within an infected individual^8,9^. As listed in Table 2, this group of identical sequences is a putative founder of each transmission cluster and only one mutation away (G5572T (NSP3-M951I) nonsynonymous mutation for cluster 6 and C26681T synonymous mutation for cluster 7) from their most recent common ancestor sequence (Fig. 4a, blue circle). The independent and random evolution patterns observed with the star-like phylogeny suggested allopatric speciation or diversification under geographic isolation. This was plausible given that most cases from the hospital occurred within the long-term care unit, which was isolated from the rest of the complex. Thus, the initial transmission of the two clusters may have occurred simultaneously at the hospital, based on the following observations: (i) the founder strains of the two clusters were one base substitution only from their most probable common ancestor; (ii) the common ancestor was not identified, despite the abundance of the two founders; and (iii) the hospital had an isolated setting.

Additionally, the church cluster (C6) and hospital cluster (C7) were unique, sharing common markers from the Wuhan-Hu-1 strain (two nonsynonymous mutations, G11083T (NSP6-L37F) and G26144T (ORF3a-G251V), and one synonymous mutation, C14805T), as presented in Table 2. As of May 8, 2020, no sequences within the two clusters were identical to any of the those registered in GISAID. Moreover, no GISAID sequences were identical to that of the most probable ancestor. However, we were able to identify a pair of identical strains that shared two common mutation markers (Fig. 4a, red circle) with this ancestor. These strains (GISAID access numbers: EPI_ISL_410546 and EPI_ISL_412974) were isolated from individuals who traveled from Wuhan to Italy, suggesting that Wuhan may have been the origin of the church and hospital clusters (see Supplementary Fig. S1).

The church cluster (C6) was a likely transmission origin of the other outbreaks that occurred outside Daegu, displaying genomic linkage with cases in Gwagju cluster (Case 2397, 8 total), Busan cluster (Case 2058, 4 total), and Gyeongsangbuk-do cluster (Case 7239, 3 total). Similarly, our genomic investigation suggested that multiple sporadic cases, such as Case 2697 in Gangwon-do and Case 4327 in Gyeonggi-do, could also be related to the church cluster (cluster 6).

## Discussion

Genomic investigations can compensate for the limitations of epidemiological contact tracing. First, this method can identify hidden relationships among new cases and previous clusters. Epidemiological contact tracing was unable to find a plausible transmission source for case 6303, an individual who resides in Seoul. However, the genomic analyses in our study suggested that this case shared a possible ancestor with cluster 2. Second, genomic investigations can provide clarity in the event that there are multiple possible transmission scenarios. Case 2058 and 3495 had travelled to China and thus they might be the potential transmission source of the church cluster (C6). However, we estimated that these cases were not a transmission origin of the church cluster, given that the strain of these two cases had one respective additional mutation than the founder strain of cluster 6. Finally, this method could mitigate human bias and possibly override human error. For example, cases 9234 and 6712 were presumed to be within the church cluster as they were both church members who had traveled to Daegu. However, we observed that case 9234 belonged to cluster 5, whereas case 6712 belonged to cluster 2, suggesting that these individuals were infected from other sources prior to their visit to Daegu; additional tracing efforts may have revealed new information regarding the spread.

By overcoming the limitations of epidemiological contact tracing, our genomic investigation could lead to new outlooks regarding the spread of COVID-19. One key observation of our study was the incongruous spread throughout Seoul, specifically regarding the newly identified members of cluster 2. Epidemiological tracing records initially identified 15 close-contact cases associated with cluster 2 in Seoul and three of these close-contact cases were sequenced in this study (cases 4023, 5431, and 3542). However, case 6303, as described above, had sequence that was identical to that of cases 5431 and 3542, strongly suggesting that case 6303 and its 12 other related cases reported by contact tracing may belong to cluster 2. Moreover, cases 9273, 4536, 2314, and 6712 were also categorized into cluster 2 by genomic sequencing, as discussed above. Thus the size of this cluster becomes double, compared to the size estimated by contact tracing. These newly discovered connections suggested that there could be another unaddressed transmission source that could have caused the various outbreaks in Seoul.

In summary, our genomic investigation of the COVID-19 outbreak in South Korea was highly consistent with our comprehensive and rigorous contact tracing records. Furthermore, genomic investigation revealed missing linkages among individuals who had not been initially traced. By providing more insight into the source and direction of transmission, genomic investigation can help public health officials implement more targeted and specific policies that prevent the spread of the disease at minimal cost to society.

## Methods

### Ethical considerations

This study has been approved by the Institutional Review Board at the Korea Centers for Disease Control and Prevention (2020-03-01-P-A) and is considered to be a public health’s act to the outbreak. Thus, the board has waived the requirement for written consent as outlined in the Title Laboratory Respondence to COVID-19. All the methods presented in this study were conducted in accordance with the relevant guidelines and regulations.

### Contact tracing and isolation

Following the strict regulations set forth by the Infection Disease Control and Prevention Act, the South Korean government created a comprehensive travel record of those with confirmed cases by collecting data via in-person questionnaire surveys and cross-checking with phone GPS traces, medical records, closed-circuit television, and credit-card transactions^10^. All asymptomatic close contacts (within 2 m) and potentially exposed individuals (within the same relative area) were mandated to self-quarantine for 14 days and were monitored twice a day by public health workers to check for the presence of fever or respiratory symptoms. A smartphone-based self-assessment application was implemented to further monitor the development of COVID-19 symptoms.

### Specimen collection and processing

Remnant specimens from COVID-19 test-positive cases in the form of nasopharyngeal and oropharyngeal (NP/OP) swabs and sputum were used for SARS-CoV-2 RNA isolation^5^. RNA was extracted from 140 µL of these specimens using a Qiagen Viral RNA Mini kit (Qiagen, Hilden, Germany) following the manufacturer’s protocol^11^.

Viral culture of SARS-CoV-2 was conducted in a Biosafety Level-3 facility according to the laboratory guidelines of Korea Centers for Disease Control and Prevention^7^. NP/OP swab specimens were inoculated into Vero E6 cells, and the cells were then cultured at 37 °C in an atmosphere containing 5% CO_2_ with 1× Dulbecco’s modified Eagle’s medium supplemented with 2% fetal bovine serum and penicillin–streptomycin. Virus replication and isolation were confirmed through cytopathic effects and gene detection by real-time reverse transcription polymerase chain reaction (RT-PCR).

### SARS-CoV-2 whole genome sequencing

In total, 10–100 ng of the extracted viral RNA with a maximum volume of 8.5 µL was subjected to target enrichment using a Truseq RNA library prep for enrichment (Illumina. San Diego, CA, USA) and Truseq RNA Enrichment (Illumina). The enriched products were size-selected and evaluated for quality and concentration using a D1000 screen tape (Agilent Technologies) on a TapeStation 4200 (Agilent Technologies). The selected libraries were quantified using a Library Quantification Kit (KAPA Biosystems) on a Quantstudio 6 Flex Real-Time PCR System (ThermoFisher Scientific). The final loading concentration was 4–14 pM, with PhiX. High-throughput sequencing was performed on a MiSeq and NextSeq 500 sequencer with 2 × 150 base pairs (Illumina).

Dual-index filtering and adapter trimming were conducted on the sequences using our in-house scripts. For quality control, low-quality bases were removed by FaQCs software with the Q_30_ threshold. The NCBI Bacteria RefSeq and Human GRCh38 databases were used to remove host reads. Filtered reads were then aligned to the reference genome sequence of Wuhan-Hu-1 (NCBI Reference Sequence: NC_045512, GenBank accession number: MN908947.3) using Burrows-Wheeler Aligner^6^. SAMtools and VCFtools were used to generate the SARS-CoV-2 whole genome consensus sequence of each specimen.

Hybridization probes were designed to cover the whole genome of SARS-CoV-2 using the Wuhan-Hu-1 strain^6^. The biotinylated probes were 120 bp in length with 3× tiling (Celemics). In total, 745 conserved probes were generated for target enrichment during sequencing library preparation.

### Maximum likelihood tree analysis

Maximum likelihood trees were generated using the PHYML program, which simultaneously adjusted the tree structure and branch length^12^. The general time-reversible model was used with the ‘ML’ option, and invariable sites were estimated with 12 substitution rate categories. The branch support values were estimated using the SH-like likelihood ratio test in PHYML3.3^13^. The tree was generated by the BIONJ option and presented using FigTree.

### Analysis of global SARS-CoV-2 strains

In total, 16,508 full-length SARS-CoV-2 sequences that were registered by May 7, 2020 were downloaded from GISAID (Supplementary Table S2)^14,15^. The sequences were globally aligned using MUSCLE^16^, and sequences at both the 5′- and 3′-ends were trimmed, yielding a 597–29,340 segment of the Wuhan-Hu-1 reference strain. Sequences shorter than this region and those with one or more ambiguous bases were filtered out, yielding 7645 fully aligned SARS-CoV-2 sequences.

## Supplementary Information

Supplementary Table S1.

Supplementary Table S2.

Supplementary Figure S1.

## Data Availability

The SARS-CoV-2 whole genome sequences described are available in GISAID (Accession IDs EPI_ISL_407193, 412869, 412870–412873, 425117, 425118, 426163, 426164, 426166, 426168, 426169, 426171, 426173, 426180–426183, 426187, 427809–427813, 471425, 471426, 471438–471455, 481370–481379, 497951–497961, 497968–497971, 497974).

## Code availability

Pre-existing software was used for our analysis.
