# Supplementary material for: Genomic investigation of the coronavirus disease-2019 outbreak in the Republic of Korea
Source: Sci Rep. 2021 Mar 16;11:6009. doi: 10.1038/s41598-021-85623-6 (PMC7971034; doi:10.1038/s41598-021-85623-6)
Supplement: Supplementary file 3 — Supplementary Figure S1. [file 41598_2021_85623_MOESM3_ESM.pdf]

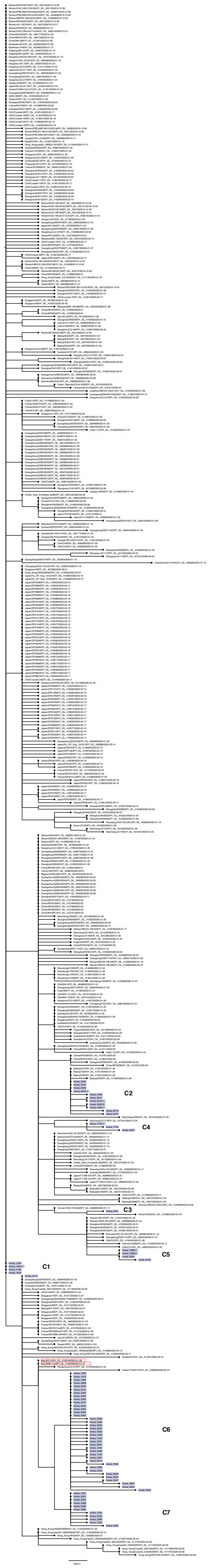
C2
C4
C3
C5
C1
C6
C7

Supplementary Figure S1 Maximum likelihood tree of all 66 cases with 408 global SARS-CoV-2 sequences collected before the church outbreak. The seven clusters we identified in this study are colored by blue. The pair of identical strains that are most close to clusters 6 and 7 are colored by red. These strains (GISAID access numbers: EPI_ISL_410546 and EPI_ISL_412974) were isolated from individuals who traveled from Wuhan to Italy.
